# Metabolic effects of prolactin and the role of dopamine agonists: A review

**DOI:** 10.3389/fendo.2022.1002320

**Published:** 2022-09-30

**Authors:** Polly Kirsch, Jessica Kunadia, Shruti Shah, Nidhi Agrawal

**Affiliations:** ^1^ New York University (NYU) Grossman School of Medicine, NYU Langone Health, New York, NY, United States; ^2^ Department of Medicine, NYU Langone Health, New York, NY, United States

**Keywords:** prolactin, hyperprolactinemia, prolactinoma, metabolic syndrome, pituitary, metabolic dysfunction, dopamine agonist

## Abstract

Prolactin is a polypeptide hormone that is well known for its role in reproductive physiology. Recent studies highlight its role in neurohormonal appetite regulation and metabolism. Elevated prolactin levels are widely associated with worsening metabolic disease, but it appears that low prolactin levels could also be metabolically unfavorable. This review discusses the pathophysiology of prolactin related metabolic changes, and the less commonly recognized effects of prolactin on adipose tissue, pancreas, liver, and small bowel. Furthermore, the effect of dopamine agonists on the metabolic profiles of patients with hyperprolactinemia are discussed as well.

## Prolactin physiology

Prolactin is a polypeptide hormone best known for its role in lactation and breast development. It is primarily produced by lactotrophs in the anterior pituitary, which make up 15-20% of the total number of pituitary cells in both males and females (1, 2). Prolactin is regulated *via* negative feedback from dopamine and also acts in a self-regulatory manner by promoting dopamine release (3, 4). Dopamine is secreted by neurons in the arcuate nucleus of the hypothalamus and acts on dopamine receptors (D_2_) in the anterior pituitary to inhibit prolactin synthesis and secretion. It was previously thought that prolactin was only produced by lactotrophs in the anterior pituitary, but recent literature indicates prolactin is also produced by the central nervous system (CNS), adipose tissue, immune system, uterus, mammary glands, and prostate gland (3, 5). The presence of prolactin receptors in nearly all organs suggests complex systemic effects of prolactin far beyond its role in reproduction, reclassifying it as a unique circulating hormone with autocrine, paracrine, and endocrine effects.

During pregnancy and lactation, prolactin plays an important role in systemic signaling. It leads to maternal adaptations that help meet the increased metabolic demands of pregnancy (6, 7). High prolactin levels promote food intake, weight gain, leptin resistance and the insulin resistant state seen in pregnancy which allows glucose to be diverted to the developing fetus (1, 7–11). These metabolic effects of prolactin are crucial to maintain metabolic homeostasis of the mother-fetus unit. This review summarizes the effects of prolactin on metabolism in the non-pregnant state.

## Prolactinoma

While there are many causes of elevated prolactin levels, prolactinomas are the leading cause of hyperprolactinemia (**Table 1**) (17, 20). Prolactinomas arise from lactotroph cells of the anterior pituitary and make up about 40% of all pituitary adenomas. Ninety percent of these tumors are less than 1 cm and are categorized as microadenomas, and the other 10% are greater than 1 cm and are considered to be macroadenomas (3, 12). Although older guidelines suggest higher cut-offs (>250 ng/mL) to diagnosis prolactinomas, newer studies highlight the ratio of prolactin to tumor volume and lower levels, between 55-94 ng/mL, may also suggest prolactin secreting pituitary tumors (13, 35–37). This distinction is important to keep in mind when differentiating prolactin secreting tumors from other causes of hyperprolactinemia.

**Table 1:** Causes of Hyperprolactinemia.

Physiological		Pathological		Pharmacological		Lab Error	
etiology:	Mechanism:	etiology:	Mechanism:	etiology:	Mechanism:	etiology:	Mechanism:
Pregnancy	• PRL levels progressively increase throughout the course of a normal pregnancy (6, 7)	Prolactinomas	• PRL secreting pituitary adenoma (12–14)	Antipsychotics	• FGAs are more likely to cause hyperPRL due to higher affinity for D2R • SGAs have a higher affinity for 5HT2A and lower affinity for D2R resulting in less hyperPRL (with the exception of risperidone, paliperidone, and amisulpride) (15, 16)	Macroprolactin	• Most PRL is monomeric, however there are larger isoforms (i.e. PRL-IgG, PRL-IgA complexes) known as macroprolactin • Macroprolactin can cross react with PRL in commercial immunoassays and cause incorrect hyperprolactinemia diagnoses (12, 13, 17)
Lactation	• Suckling induces PRL release (2, 7)	Hypothalamic and Pituitary Stalk Disorders	• Lesions that compress the pituitary stalk can interrupt dopamine inhibition of PRL release (ex: NFPA, Rathke's cyst, craniopharyngioma, meningioma, etc.) (13, 14, 18, 19)	Antidepressants and Anxiolytics	• Affect on Serotonergic pathways can lead to mild increase in PRL (20, 21)		
Ovulation	• The high estrogen state seen during ovulation can cause increased PRL release (7, 17)	Primary Hypothyroidism	• High TRH stimulates lactotroph cells to secrete PRL (22)	Antiemetics	• Antagonize D2R, causing hyperPRL (Domperidone, Metoclopramide) (21).		
Stress	• Mechanism is not well understood • Stress may induce changes in dopamine and serotonin, increasing PRL release (23, 24)	Chronic Renal Failure	• Combination of decreased PRL excretion and increased PRL secretion (25)	Opioids	• μ-, κ- and δ- opioid receptor mediated hyperPRL (26)		
Chest Wall Injury	• Likely a similar mechanism to suckling (13, 27)	Cirrhosis	• Decrease in dopamine inhibition of PRL and increase in estrogen (28)	Antihypertensives	• Verapamil (non-dihydropyridine calcium channel blocker) suppresses dopamine and can cause hyperPRL (29)		
Exercise	• Mechanism is not well understood • PRL may be elevated for about 30 minutes after high intensity exercise (13, 30)	Polycystic Ovarian Syndrome	• Mechanism is not well understood • Elevated PRL is often seen in PCOS (31, 32)				
		Seizures	• Mechanism is not well understood • There may be a transient increase in PRL release 10-20 minutes post seizure (33, 34)				

PRL, prolactin; NFPA, non-functioning pituitary adenoma; TRH, Thyrotropin-releasing hormone; PCOS polycystic ovarian syndrome; FGA, first generation antipsychotic; SGA second generation antipsychotic; 5HT2A, Serotonin 5HT2A Receptor; D_2_R, Dopamine Receptor D_2_; hyperPRL, hyperprolactinemia.

**Figure 1:**
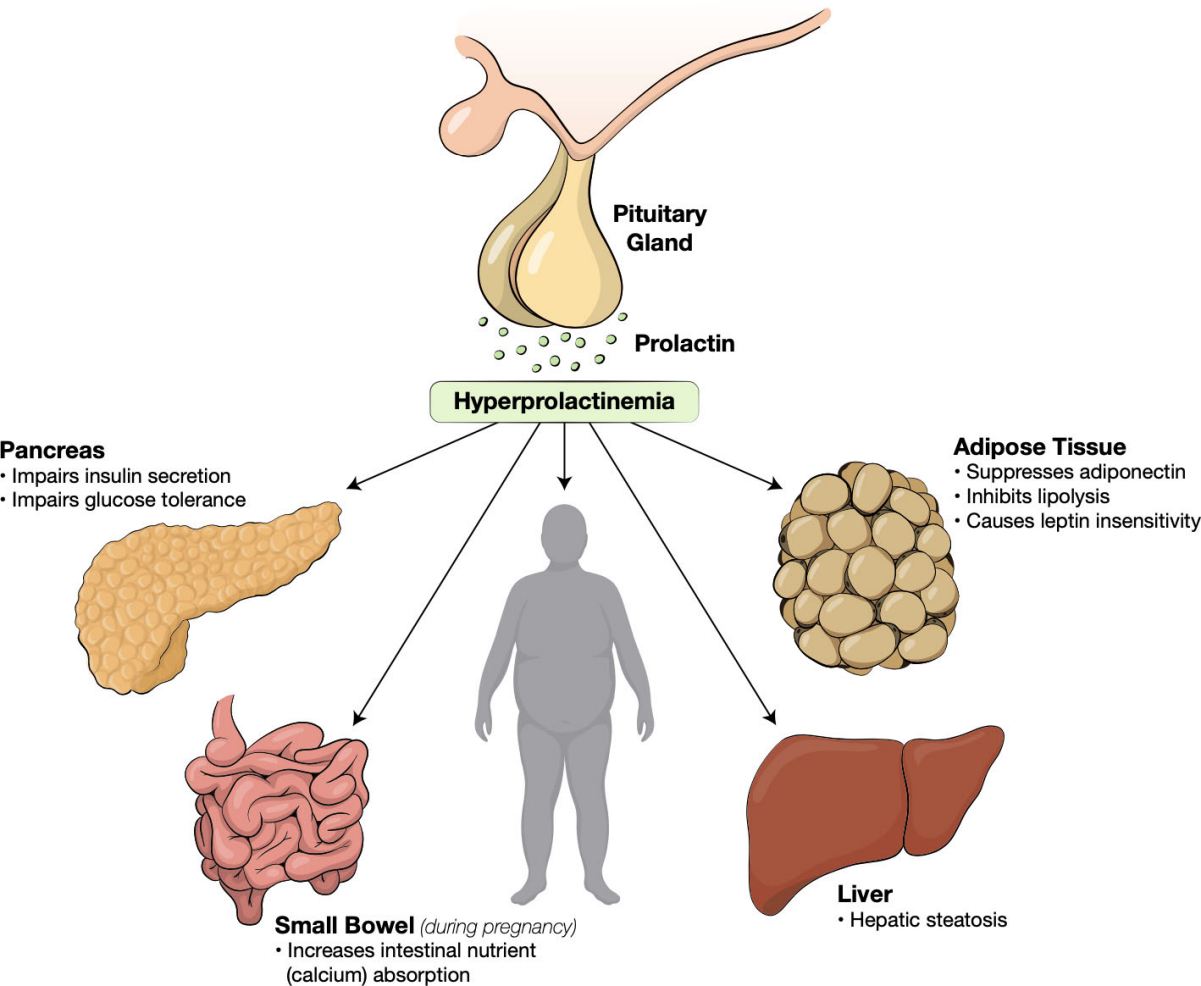
Metabolic effects of hyperprolactinemia.

Prolactinomas are most often seen in female patients between the ages of 20 to 40. Young females may experience symptoms of galactorrhea and amenorrhea. Males may experience impotence and erectile dysfunction, but often present later in their course with headaches and visual deficit secondary to mass effects from larger tumors (3). Regardless of the cause, hyperprolactinemia is strongly correlated with widespread metabolic alterations including obesity, insulin resistance, dyslipidemia, non-alcohol fatty liver disease (NAFLD) and endothelial dysfunction (**Figure 1**) (6, 38–40).

**Figure 2:**
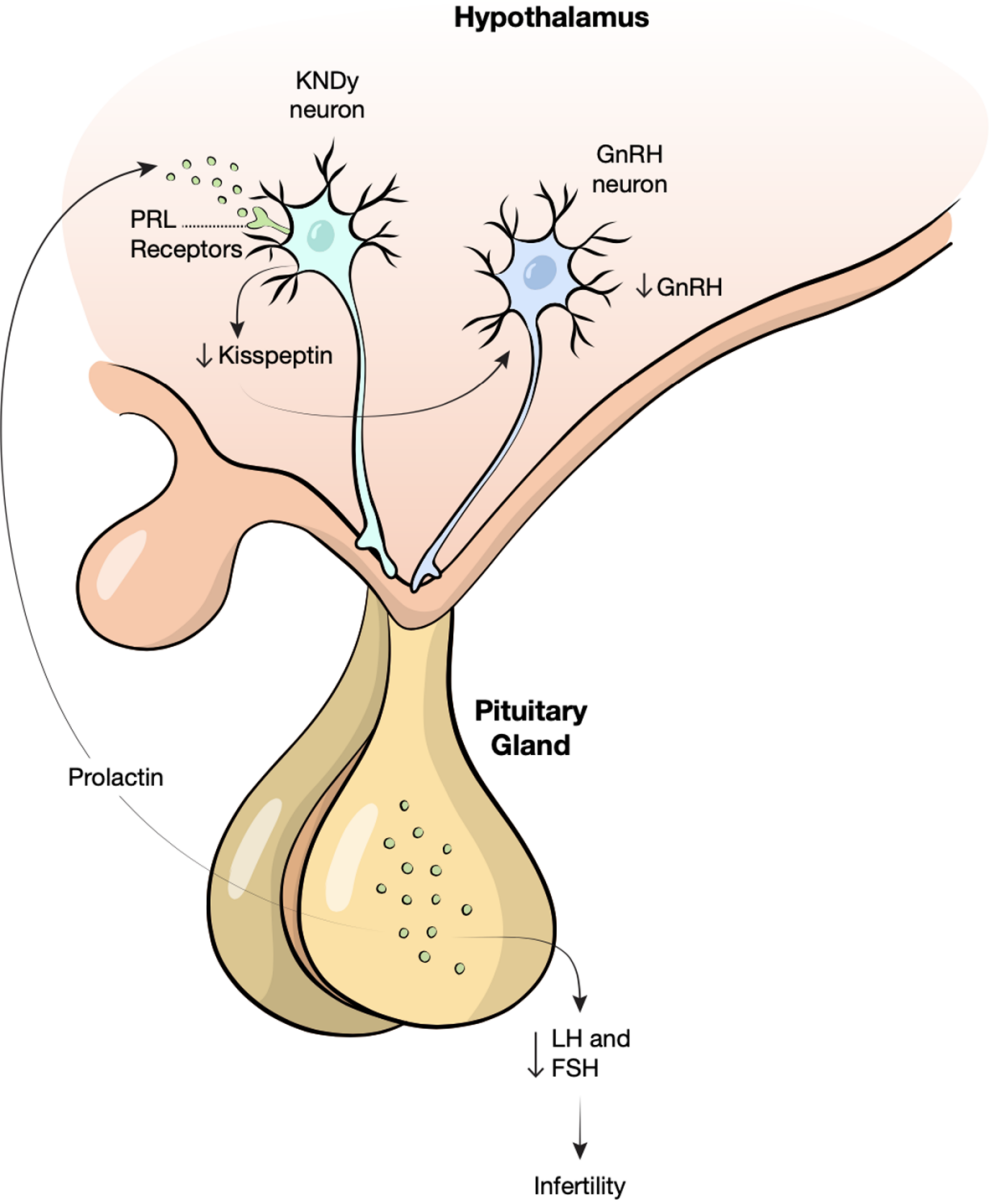
Proposed mechanism of hyperprolactinemia induced infertility. Prolactin (PRL) binds receptors on kisspeptin, neurokinin B, and dynorphin neurons (KNDy) in the hypothalamus, decreasing the release of Kisspeptin. Low kisspeptin may cause loss of gonadotropic releasing hormone (GnRH) pulsatility resulting in decreased follicle stimulating hormone (FSH) and luteinizing hormone (LH) and therefore infertility.

The pathophysiology of hyperprolactinemia and infertility is complex; there is significant crossover of the neuropeptides involved in the regulation of fertility and metabolism. In women, elevated prolactin levels are thought to cause infertility by suppressing pulsatile gonadotropin releasing hormone (GnRH) release that is necessary to induce luteinizing hormone (LH) and follicle stimulating hormone (FSH) secretion and ovulation (41). The pulsatile release of GnRH is stimulated by a neuropeptide called kisspeptin (17). Kisspeptin is secreted by the kisspeptin, neurokinin B, and dynorphin neurons (KNDy) in the hypothalamus. KNDy neurons express prolactin receptors and hyperprolactinemia causes a decrease in kisspeptin secretion hence decreasing pulsatile GnRH release (**Figure 2**) (41). The therapeutic role of kisspeptin in patients with hyperprolactinemia-induced infertility has recently been studied. Kisspeptin may be used to stimulate GnRH secretion and restore ovarian function (42, 43).

Kisspeptin also exerts metabolic effects both directly *via* receptors in the brain, pancreas and adipose tissue and indirectly though the GnRH reproductive axis pathway (44). Kisspeptin synthesis and release is regulated by metabolic factors, such that it is only secreted in situations of energy surplus to sustain reproduction (45, 46). Several animal studies support the theory that kisspeptin may be involved in regulating insulin secretion and lipid metabolism, suggesting there may be a role of kisspeptin in the treatment of these metabolic derangements (47–49).

## Metabolic effects of prolactin

### Neurohormonal appetite regulation

**Figure 3:**
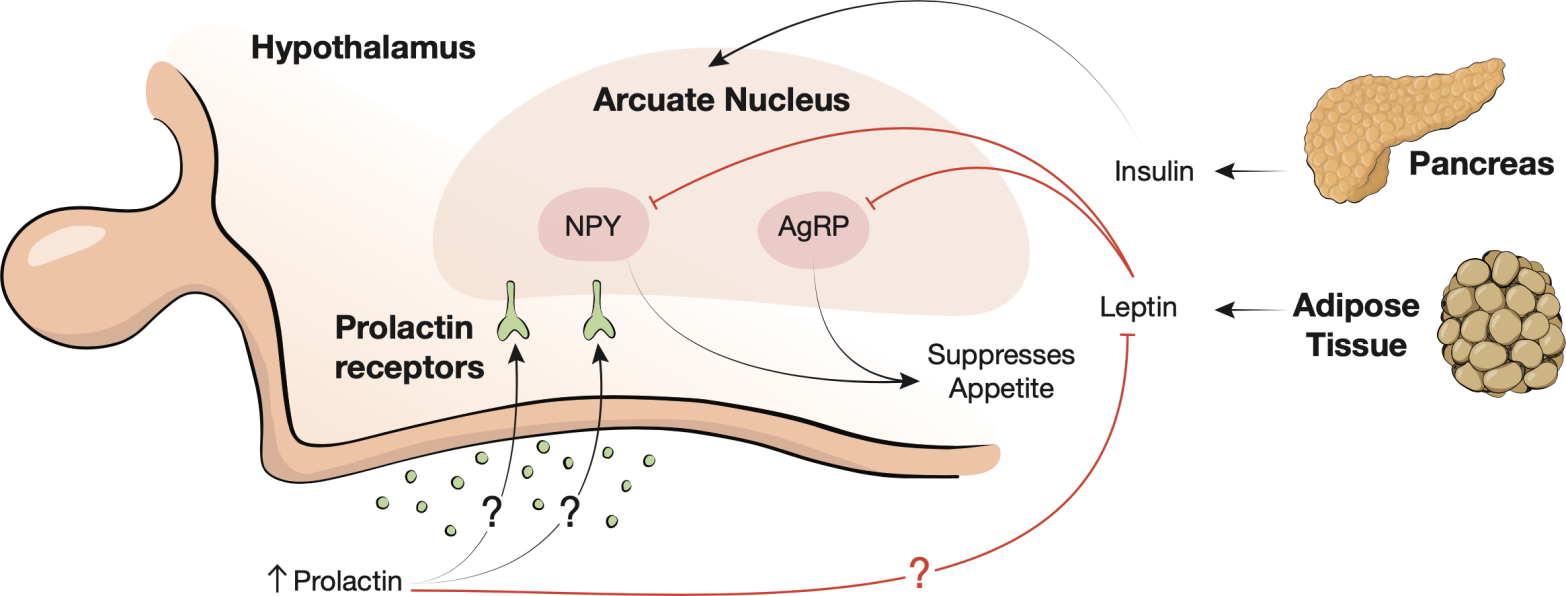
Neurohormonal appetite regulation. The arcuate nucleus of the hypothalamus integrates signals from insulin, leptin, and circulating nutrients. Leptin is secreted by adipose tissue and suppresses agouti-related protein (AgRP) and neuropeptide Y (NYP) neurons (red inhibition lines) to suppress appetite. Prolactin may also bind to the arcuate nucleus receptors leading to leptin insensitivity (red inhibition line).

Prolactin plays a significant role in the neurologic regulation of appetite and satiety (**Figure 3**). The arcuate nucleus of the hypothalamus, and specifically, anorectic pro-opiomelanocortin (POMC), orexigenic neuropeptide Y (NYP), and agouti-related peptide neurons (AgRP) are key players in the regulation of appetite (6, 50). The arcuate nucleus integrates signals from circulating nutrients and hormones such as leptin and insulin to maintain metabolic homeostasis (51). Leptin is an adipokine secreted in direct proportion to the both the size and quantity of adipocytes in the body; it suppresses NYP and AgRP neurons at the arcuate nucleus, thereby suppressing appetite (52, 53). Prolactin may influence the hypothalamic appetite pathway in different ways. Though the molecular mechanism remains elusive, prolactin may exert influence on the arcuate nucleus indirectly by causing leptin insensitivity (54). Chronically elevated prolactin can create a pseudopregnancy state with resultant hyperphagia and weight gain (55, 56).

Additionally, disruption of CNS dopaminergic tone with relative dopamine deficiency may lead to hyperphagia. As described above, prolactin self regulates by increasing dopamine release (4). In high prolactin states, central dopaminergic neurons become refractory to prolactin, contributing to weight gain and insulin resistance (7, 38, 57). Therefore, in addition to prolactin’s potential influence on eating behaviors through leptin insensitivity, prolactin may further stimulate hyperphagia by decreasing dopaminergic tone (7). Patients with polycystic ovarian syndrome (PCOS) have low hypothalamic dopaminergic tone leading to inappropriate prolactin and LH secretion. Ironically, high prolactin levels in this disease state can be associated with improved metabolic profiles (31, 32, 58, 59). However further discussion of PCOS related hyperprolactinemia is outside the scope of this article (32, 59, 60).

### Adipose tissue

At physiologic levels (male 2-18 ng/mL, female 2-30 ng/mL), prolactin is important in maintaining adipogenesis and adipocyte differentiation. It is secreted by human adipocytes and has autocrine effects causing decreased activity of lipoprotein lipase and inhibiting lipolysis (38). Kok et al. demonstrated that obese women have higher prolactin secretion compared to healthy weight controls (61). They found that spontaneous prolactin release is increased in obese women, proportional to both body mass index (BMI) and visceral fat likely linked to decreased D_2_ activity (61). While it is recognized that significantly elevated prolactin levels are associated with weight gain, obesity, and overall metabolic dysfunction, the metabolic effects of prolactin vary even within a physiologic range (6, 38, 62–66). A recent study by Liu et al. categorized patients as metabolically healthy obese (i.e. BMI ≥ 30 with normal triglycerides, high density lipoprotein (HDL), blood pressure, and fasting blood glucose), metabolically unhealthy obese (i.e. BMI ≥ 30 with abnormal triglycerides, HDL, blood pressure, or fasting blood glucose), and controls (67). They found that metabolic healthy obese patients had slightly higher, although still physiological, prolactin levels (19.9 ng/mL) compared to metabolically unhealthy obese patients (14.4 ng/mL) and controls (12.4 ng/mL). These findings suggest that normal prolactin levels may serve as a regulatory mechanism, favoring energy metabolism in the obese state (67, 68).

Prolactin is also involved in metabolism through its effect on adiponectin, a hormone secreted by adipose tissue. Low adiponectin levels are associated with insulin resistance, obesity, and atherosclerosis (69). A study on adiponectin during pregnancy suggests that prolactin likely suppresses maternal adiponectin, thereby contributing to the new homeostatic set point characteristic of pregnancy (69). Although there have not been definitive studies demonstrating the same role for adiponectin in the non-pregnant state, animal studies have shown that adiponectin secretion is significantly suppressed by prolactin in mice *in vivo* and in human adipose tissue *in vitro* (70).

### Pancreas

Prolactin acts to modulate beta cell growth and insulin resistance through direct action on the pancreas (71, 72). In fetal life, prolactin is important in pancreatic islet cell development. It has been shown that prolactin receptor deficient mice have reduced beta cell mass and lower insulin content, implying that prolactin plays a key role in developing beta cells during embryogenesis and gestation (7, 73, 74). In addition to dopamine’s role in regulating prolactin, dopamine impacts glucose homeostasis through a variety of mechanisms that are entirely independent of prolactin. Dopamine receptors (D_2_ and D_3_) found in the pancreas play a role in regulating insulin secretion (75). Blocking dopamine receptors can result in glucose intolerance, hyperinsulinemia, and weight gain. In fact, bromocriptine which is FDA approved for use in patients with type 2 diabetes, is a dopamine agonist that increases insulin sensitivity and decrease glycogen formation (76). Bromocriptine is effective in reducing hemoglobin A1C by 0.4-0.8% and improving hyperlipidemia and obesity, thereby decreasing cardiovascular risk by up to 40% (77, 78).

The concentration of prolactin influences the relationship between prolactin and insulin resistance. A study by Wagner et al. with participants between ages 18-80, found that over the age of 29 higher prolactin levels were associated with insulin sensitivity and lower glucose levels (AUC, P<0.0005) (79). It is hypothesized that this relationship may be due to differences in dopaminergic tone in the CNS and its effect on peripheral metabolism (79). Although causality is hard to predict and larger studies are needed, this suggests an age-dependent relationship of prolactin and insulin sensitivity (79). Park et al. found that in diabetic mice, beta cell expansion is stimulated by both low (25 µg/kg bw/12 h) and high (250 µg/kg bw/12 h) dose prolactin injections (80). At the end of the experimental period, mice receiving low dose prolactin injections had a mean prolactin level of 43.8 +/- 4.2 ng/mL and improved hepatic insulin sensitivity and insulin secretion. In contrast, mice receiving high prolactin doses had a mean prolactin level of 205.3 +/- 6.8 ng/mL with increased insulin resistance and impaired glucose tolerance (80, 81). The effect of treatment with prolactin lowering drugs, i.e. dopamine agonists, on various metabolic parameters is discussed in section 4.

### Liver

At physiologic levels, prolactin may have a protective role in the liver. Prolactin receptors are expressed widely throughout the liver. Prolactin acts on these receptors to prevent hepatic steatosis by decreasing triglyceride accumulation (6, 82, 83). The CD36 fatty acid transporter is central in the pathogenesis of hepatic steatosis. Increased hepatic prolactin receptor expression is associated with decreased CD36 gene expression, thereby protecting against the development of hepatic steatosis (84). Zhang et al. found that prolactin levels are lower in patients with NAFLD compared to healthy controls (84). Furthermore, prolactin levels were found to be lower in patients with severe hepatic steatosis when compared to patients with mild to moderate disease (84). This does not hold true in pathological hyperprolactinemia secondary to pituitary adenomas. In these patients with significantly elevated prolactin, the protective effects of the hormone disappear. Patients may develop increased triglyceride and lipid droplet accumulation, favoring development of hepatic steatosis [25]. The underlying mechanism of this is poorly understood.

In addition to its role in hepatic steatosis, prolactin has been shown to impact insulin sensitivity. Prolactin improves hepatic insulin sensitivity by inducing signal transducer and activator of transcription 5 (STAT5) phosphorylation (6). Prolactin receptor expression is increased in insulin sensitive states and decreased in insulin resistant states both *in vitro* and *in vivo*. Supporting this proposed mechanism, it has been shown that hepatic prolactin receptor knock-out mice display impaired glucose tolerance and worsening insulin resistance (85).

### Small bowel

Though not classically considered to be a metabolic organ, the small bowel is essential for nutrient absorption and therefore can impact weight gain and metabolism. Throughout pregnancy and lactation, prolactin is thought to act on receptors expressed throughout epithelial cells of the small intestine. A study in rodents showed that prolactin causes the small bowel surface area to increase, through both increasing height of the villi and length of the small intestine to allow for maximum nutrient absorption (86, 87). Though this is a compelling theory, studies on this topic are conflicting (88). Interestingly, prolactin’s effect on the intestinal mucosa has been specifically shown to increase intestinal calcium absorption in rats *in vivo* and human tissue *in vitro* which further supports the demands of pregnancy and lactation (89). Little research has been conducted on the influence of prolactin on the small bowel outside of the context of reproduction.

## Metabolic effects of dopamine agonists in patients with hyperprolactinemia

It is well established that hyperprolactinemia causes metabolically deleterious effects (6, 38). Dopamine agonists bromocriptine (BRC) and cabergoline (CAB) are usually first line, though some patients require pituitary surgery and rarely radiation therapy to control their hyperprolactinemia. **Table 2** summarizes seminal studies evaluating the metabolic effects of dopamine agonists in patients with hyperprolactinemia. We reviewed 17 studies from 2002 to 2021 that looked at changes in metabolic parameters including BMI, low density lipoprotein (LDL), and homeostatic model assessment for insulin resistance (HOMA-IR) (90–106). The average sample size was 28 and 11 of the studies reviewed were prospective. Across all studies, the mean age of patients at baseline was 35.1 years. Approximately 51% of the patients studied were female and 43% of tumors were macroprolactinomas. All 17 studies used a dopamine agonist as treatment (10 used CAB only, 6 used BRC or CAB, 2 used BRC only), and in 2 of these studies pituitary surgery was used in addition to medical therapy. No notable differences were seen between the 2 studies that used surgery in addition to medical treatment compared to those that used medical treatment alone, indicating that these results were independent of the modality of treatment.

**Table 2:** Summary of recent studies evaluating the effects of dopamine agonists on metabolic parameters.

Study	Year	Sample Size	Age, mean +/- SD	Female %	Macroprolactinoma %	Treatment	Drug doses	Baseline PRL ng/mL	PRL Format	Months to Post Tx Follow-Up	Baseline BMI	Post Tx BMI	BMI Format	P value BMI Baseline vs Post Tx	Baseline LDL	Post Tx LDL	LDL Format	P value LDL Baseline vs Post Tx	Baseline HOMA-IR	Post Tx HOMA-IR	P value HOMA-IR Baseline vs Post Tx	HOMA-IR Format
**Posawetz** (90)	2021	12	40 +/- 17	33	57	Cabergoline	0.25-1.5 mg/wk	5270 (2234–15374)	median (IQR)	2.3	27.5 (22.4-33.5)	27.5 (21.5-35)	median (IQR)	0.686	130 (107-147.5)	106.5 (94.3-148)	median (IQR)	0.018^§^	2.32 (1.64–3.59)			median (IQR)
**Pirchio** (91)	2021	34	33.9 +/- 12.5	35	88	Cabergoline, pituitary surgery	0.25-7 mg/wk	15313 +/- 49864	mean +/- SD	12					Surg + CAB = 124.14 +/- 28.4 CAB = 123.6 +/-24.43	Surg + CAB = 112.36 +/- 25.57 CAB = 115.33 +/-38.01	mean +/- SD	Surg + CAB: 0.196 CAB: 0.301	Surg + CAB = 3.37 +/- 2.01 CAB = 4.84 +/-5.8	Surg + CAB = 3.16 +/- 1.68 CAB =3.8 +/- 4.63	Surg + CAB = 0.33 CAB = 0.055	mean +/- SD
**Andereggen** (92)	2021	30	48 +/- 12.6	43	67	Cabergoline, Bromocriptine, pituitary surgery	CAB: 0.5-2 mg/wk BRC: 2.5-10 mg/d	18213 (3277–137723)	median (IQR)	51.9	28.6 +/- 6	26.5 +/-6	mean +/- SD	0.05*	139.21 +/- 30.94	131.48 +/- 38.67	mean +/- SD	0.07				
**Khalil** (93)	2021	32	36.09 +/- 9.54	44	47	Cabergoline , Bromocriptine	5.8 +/- 4.1 mg/d (range 1.25-15 mg)	130198 +/- 24849	mean +/- SD	3	28.9 +/- 4.28	24.53 +/- 2.2	mean +/- SD	<0.0001*	126.96 +/- 18.66	92.93 +/- 5.57	mean +/- SD	<0.0001^§^	1.34 +/- 0.17	0.94 +/- 0.13	<0.0001^†^	mean +/- SD
**Schwetz** (94)	2017	53	39 +/- 17	42	59	Cabergoline	0.5 mg/wk (IQR 0.5–0.9 mg)	7607 +/- 4414	mean +/- SD	9	27.9 +/- 5.9	28.6 +/- 5.6	mean +/- SD	0.396	121.6 +/- 39.4	110.6 +/- 37.6	mean +/- SD	0.005^§^				
**Auriemma** (95)	2015	32	42 +/-5	0	78	Cabergoline	0.25-3.5 mg/wk	42996 +/- 93443	mean +/- SD	12, 24	31.7 +/- 3.9	12 mos: 30.4 +/- 3.6 24 mos: 29.3 +/- 3.4	mean +/- SD	12 mos: 0.000* 24 mos: 0.017*	142.3 +/- 30.94	12 mos: 120.26 +/- 23.2 24 mos: 123.74 +/- 30.94	mean +/- SD	12 mos: 0.001^§^ 24 mos: 0.006^§^	4.1 +/- 2.2	12 mos: 2.7 +/- 1.2 24 mos: 1.8 +/- 1.07	12 mos: 0.000^†^ 24 mos: 0.000^†^	mean +/- SD
**Medic** (96)	2015	20	30 +/- 7	100	30	Cabergoline, Bromocriptine	CAB: 1.08 +/- 45 mg/wk BRC: 6.07 +/- 1.83 mg/d	2919 +/- 1102	mean +/- SD	4	24.046 +/- 6.360	22.815 +/- 6.093	mean +/- SD	0.028*	128.46 +/- 46.44	102.78 +/- 29.47	mean +/- SD	0.002^§^				
**Pala** (97)	2015	19	27 +/- 6	95	21	Cabergoline	0.5 mg/wk	2514 +/- 2232	mean +/- SD	2, 6	24.2 +/- 4/0	2 mos: 23.9 +/- 4.2, 6 mos: 23.2 +/- 3.9	mean +/- SD	2 mos: 0.09 6 mos: <0.001*	108.28 +/- 34.8	2 mos: 88.94 +/- 19.33, 6 mos: 77.34 +/- 11.6	mean +/- SD	2 mos: 0.01^§^ 6 mos: <0.001^§^	1.10 (1.27)	2 mos: 1.21 (1.10) 6 mos: 1.04 (0.52)	2 mos: 0.71 6 mos: 0.064	median (IQR)
**Barbosa** (98)	2014	21			23	Cabergoline, Bromocriptine		9080 +/- 7034	mean +/- SD	6	29.6 (18.6–39.4)	28.4 (18.9–38.5)	median (min-max)	0.58	122 (75–223)	99 (66–159)	median (min-max)	<0.01^§^	1.59 (0.33-20.2)	1.33 (0.26–15.4)	0.05^†^	median (min-max)
**Auriemma** (99)	2013	61	34 +/- 10	79	33	Cabergoline	0.25-5.5 mg/wk	16733 +/- 5073	mean +/- SD	12, 60	27.6 +/- 5.3	12 mos: 26.4 +/- 4.6 60 mos: 24.3 +/- 4.7	mean +/- SD	12 mos: 0.53 60 mos: 0.000*	126.06 +/- 34.8	12 mos: 113.3 +/- 23.2, 60 mos: 97.83 +/- 27.07	mean +/- SD	12 mos: 0.085 60 mos: 0.000^§^	3.2 +/- 2.09	12 mos: 2.07 +/- 1.15, 60 mos: 1.15 +/- 0.77	12 mos: 0.002^†^ 60 mos: 0.000^†^	mean +/- SD
**Ciresi** (100)	2013	43	34 +/- 11	81		Cabergoline	0.25-1.80 mg	3715 +/- 5718	mean +/- SD	12	25.57 +/- 5.18	25.41 +/- 4.97	mean +/- SD	0.177	110.21 +/- 35.96	93.58 +/- 26.3	mean +/- SD	<0.01^§^	3.87 +/- 1.53	2.93 +/- 0.96	<0.01^†^	mean +/- SD
**Inancli** (101)	2013	21	30 +/-10	100	14	Cabergoline	0.5 mg/d	3201 +/- 1230	mean +/- SD	6	27.1 +/- 5.9	26.7 +/- 5.6	mean +/- SD	0.03*	106.2 +/- 27.1	91.7 +/- 34.1	mean +/- SD	0.01^§^	1.25 (0.22–4.5)	1.02 (0.24–4.1)	0.02^†^	median (min-max)
**Berinder** (102)	2011	14	40 +/- 14	57	43	Cabergoline, Bromocriptine	CAB: 0.5 mg/wk BRC: 1.25-15 mg/d	M: 26809, F: 1511	mean	2, 6	F: 25.1 (17.8–29.2) M: 27.6 (23.4-32.9)	F 2 mos: 25.0 (17.8–29.1), M 2 mos: 26.7 (23.4-30.9), F 6 mos: 25.3 (18.6–30.4), M 6 mos: 25.4 (22.8–29.6)	median (min-max)	6 mos: 0.046*	131.48 +/- 34.8	2 mos: 112.14 +/- 23.2, 6 mos: 112.14 +/- 23.2		2 mos: 0.003^§^ 6 mos: 0.002^§^	1.4 (0.6–9.8)	2 mos: 1.6 (0.7–4.2), 6 mos: 2.1 (0.8–3.5)	NS	median (min-max)
**Silva** (103)	2011	22	42 +/- 35	77	18	Cabergoline, Bromocriptine		5720 +/- 3621	mean +/- SD	6	29.5 (18.6–39.2)	28 (18.9-38.5)	median (min-max)	0.4759	116 (75–223)	100 (66–159)	median (min-max)	0.002^§^	1.4 (0.33-20.2)	1.3 (0.3-15.4)	0.339	median (min-max)
**Serri** (104)	2006	15	39 +/- 13	53	13	Cabergoline	0.5-2 mg/wk	20160 +/- 18780	mean +/- SD	3	29 +/- 6				110.21 +/- 29.78	105.18 +/- 32.1	mean +/- SD	0.42	4.51 +/- 1.9	4.17 +/- 1.6	0.07	mean +/- SD
**Yavuz** (105)	2003	16	31 +/- 10	0		Bromocriptine	2.5-20 mg/d	3318 +/- 1637	mean +/- SD	6	26.3 +/- 5.3	25.0 +/- 5.3	mean +/- SD	NS	123.5 +/- 30	117.3 +/- 35	mean +/- SD	NS	2.127 +/- 1.1	1.521 +/- 0.42	<0.01^†^	mean +/- SD
**Doknic** (106)	2002	23	37 +/-3	48	65	Bromocriptine	15-20 mg/d	42682 +/- 37429	mean +/- SD	1, 6, 24	F: 24.4 +/- 1.2, M: 30.4 +/- 1.7	F 1 mo: 24.1 +/- 1.2, M 1 mo: 30.2 +/- 1.7, F 6 mos: 23.1 +/- 1.0, M 6 mos: 28.6 +/- 1.6, F 24 mos: 23.6 +/- 0.8, M 24 mos: 26.5 +/- 1.9	mean +/- SD	>0.05								

SD, standard deviation; PRL, prolactin; Tx, treatment; BMI, body mass index; LDL, low-density lipoprotein; HOMA-IR, homeostatic model assessment for insulin resistant; IQR, interquartile range; d, day; wk, week; mos, months; M, male; F, female; Surg, surgery; CAB, cabergoline; BRC, bromocriptine. *p < 0.05 vs baseline for BMI. ^†^p < 0.05 vs baseline for LDL. ^§^p < 0.05 vs baseline for HOMA-IR. NS, Not Significant.

The mechanism by which prolactin levels maintain metabolic homeostasis is not clearly understood. Of the 17 studies listed in **Table 2**, 15 evaluated the effects of treatment of elevated prolactin on BMI. Of these studies, 8 found a statistically significant decrease (p < 0.05) in BMI (mean of 27.2 pre-treatment vs. 25.5 post-treatment, average decrease of 1.92) after a mean of 14.4 months of treatment (92, 93, 95–97, 99, 101, 102), while 7 did not (90, 94, 98, 100, 103, 105, 106). Of the 16 studies that reported LDL values, 12 studies found a statistically significant decrease (*p* < 0.05) in LDL (mean of 121.2 mg/dL pre-treatment vs. 100.8 mg/dL post-treatment, average decrease of 20.4 mg/dL) after a mean of 12 months of intervention (90, 93–103), while 4 did not reach significance (91, 92, 104, 105). Pala et al. found that improvement in lipid parameters is not related to weight loss or BMI, suggesting that there is likely a direct effect of dopamine agonists on lipids that is independent of weight (97). Likewise, Auriemma et al. found that weight, waist circumference, and BMI are significantly reduced after 60 months of CAB treatment (99). The prevalence of metabolic syndrome appears to decrease in a shorter different time frame, at 12 months, again supporting the idea that metabolic syndrome is at least somewhat independent of weight loss (99). Though prolactin is involved in lipid metabolism, D_2_ receptors are also present on adipose tissue, implying an independent regulatory role for dopamine (99). Ciresi et al. found no association between prolactin levels and metabolic parameters at baseline or at 12 months follow up (100). They report that the relationship between decrease in prolactin levels and improvement of metabolic parameters appears to reflect the effect of treatment with dopamine agonists, rather than prolactin normalization (100). This supports that idea that dopamine agonists offer a prolactin-independent mechanism of improving a patient’s metabolic profile. This discrepancy between improvement in BMI and LDL in the reported studies could possibly be due to the independent effects of dopamine and prolactin.

Of the 12 studies that evaluated HOMA-IR improvement in hyperprolactinemic patients treated with dopamine agonists, 7 studies found a statistically significant decrease (p < 0.05) in HOMA-IR (mean of 2.93 pre-treatment vs. 1.67 post-treatment, average decrease of 1.26) after a mean of 16.7 months of intervention (93, 95, 98–101, 103, 105), while 5 did not (91, 97, 102–104). Pirchio et al. explain that improvement in glucose profile correlates with dopamine agonist dose rather than with prolactin level (91). As discussed previously, dopamine plays a role in glucose homeostasis by binding D_2_ and D_3_ receptors in the pancreas preventing excessive sympathetic activity and hepatic glucose production, thereby improving insulin sensitivity. Additionally, it often takes greater than 12 weeks of follow-up to see improvement in HOMA-IR which would go along the normal course of dopamine agonist treatment. Analyzed together, this data provides compelling evidence that dopamine agonists have a direct effect on metabolism, beyond their role in prolactin normalization.

## Prolactin: A “goldilocks zone”

Though it is clear that high prolactin levels are metabolically unfavorable, multiple studies have also shown low prolactin levels to be associated with increased metabolic disease (107–109). For example, Manshaei et al. found that prolactin levels in diabetic patients were significantly lower (5.32 ± 0.36 ng/mL) when compared to healthy controls (18.38 ± 2.3 ng/mL) (109). There may be a “goldilocks zone” for serum prolactin that promotes metabolic homeostasis, though exact ranges are variable amongst studies (1, 110, 111). Li et al. showed that normal to high prolactin levels (median 24, range 16-35 ng/mL) were associated with lower risk of type 2 diabetes at 20 years follow up compared to women with lower prolactin levels (median 6, range 5-8 ng/mL ng/mL) (112). In a smaller case control study of 134 patients, the average concentration of prolactin in controls (18.38 µg/L) was found to be significantly higher than that of diabetic patients (5.39 µg/L) (109). Wang et al. also similarly showed that a high normal prolactin range (11.61-26.29 ng/mL) was most protective against insulin resistance and diabetes in a large cohort of patients with hyperprolactinemia (107, 113). Furthermore, as previously described, Liu et al. found data supporting the idea that normal prolactin levels are metabolically beneficial, favoring energy metabolism in the obese state (67, 68).

The metabolic effects of prolactin at the different ranges are variable. Macotela et al. suggest a scale where intermediate levels of prolactin (25-100 ng/mL) are considered metabolically favorable with least untoward consequences, whereas low-normal prolactin levels (1-5 ng/mL) and significantly elevated levels (>100 ng/mL) are considered metabolically detrimental (1). More recently, in a study of 18 to 45 year-old women on CAB, the cardiometabolic profile of patients with low prolactin levels (less than 5 ng/mL) were compared to those within the reference range of 5-25 ng/mL (110). The hypoprolactinemic patients had higher glucose levels, hemoglobin A1C, triglycerides, insulin resistance and lower levels of HDL on analysis. Although this was a small study of 16 patients with low prolactin levels, the mechanistic link between low prolactin levels and metabolic dysregulation is certainly noteworthy (110).

In several studies (discussed in section 4), prolactin lowering drugs i.e. dopamine agonists are shown to improve metabolic profiles. Although this may appear counterintuitive to the “goldilocks zone” hypothesis, it is suggested that increased dopamine and higher levels of prolactin can improve metabolic fitness through distinct mechanisms, as delineated above. In fact, there may even be a role for prolactin enhancing drugs in the treatment of metabolic disease. Animal studies show that amisulpride, a dopamine receptor antagonist which enhances prolactin levels, improves the glucose profile in obese mice (114). Future prospective studies on this topic are clearly needed.

## Conclusion

Prolactin is a unique hormone with action at various extra-pituitary sites, categorizing it as a hormone with classic, autocrine and paracrine activity. It is critical in pregnancy and lactation, during which prolactin levels rise to allow for metabolic adaptations through the cycle of reproduction but its role in metabolism is distinctive as well. Hyperprolactinemia causes a vast array of unfavorable metabolic effects but suppressed prolactin levels can also increase cardiometabolic risk. It is suggested that there may be a “goldilocks zone” where a high-normal prolactin is associated with optimal metabolic health; however the prolactin ranges are variable across studies. While some studies show improvement in BMI, LDL and HOMA-IR after prolactin normalizing treatment, the results are inconsistent. Large prospective studies with definitive metabolic end points are required to determine the most favorable prolactin level for metabolic health as well as the role for prolactin lowering and enhancing medications.

## Author contributions

PK – Manuscript drafting and editing. JK - Manuscript drafting and editing. SS – Manuscript drafting and editing. NA – Crafting of manuscript contents, oversight of manuscript drafting and editing. All authors contributed to the article and approved the submitted version.

## Acknowledgments

**Figures 1**, **2**, and **3** created by Kristen Dancel-Manning.

## Conflict of interest

Author NA is on the advisory board for Corcept Therapeutic and is a principal Investigator for institution-directed research grants (Chiasm Acromegaly Registry).

The remaining authors declare that the research was conducted in the absence of any commercial or financial relationships that could be construed as a potential conflict of interest.

## Publisher’s note

All claims expressed in this article are solely those of the authors and do not necessarily represent those of their affiliated organizations, or those of the publisher, the editors and the reviewers. Any product that may be evaluated in this article, or claim that may be made by its manufacturer, is not guaranteed or endorsed by the publisher.
